# Public willingness to adhere to COVID-19 precautionary measures in Sudan: an application of the Health Belief Model

**DOI:** 10.11604/pamj.2021.39.135.29171

**Published:** 2021-06-17

**Authors:** Azza Mehanna, Yasir Ahmed Mohammed Elhadi, Don Eliseo Lucero-Prisno III

**Affiliations:** 1 Department of Health Administration and Behavioral Sciences, High Institute of Public Health, Alexandria University, Alexandria, Egypt,; 2 Department of Public Health, Medical Research office, Sudanese Medical Research Association, Khartoum, Sudan,; 3 Department of Global Health and Development, London School of Hygiene and Tropical Medicine, London, United Kingdom

**Keywords:** COVID-19, Sudan, preventive measures, precautionary behavior, preventive guidelines, public health, health promotion

## Abstract

**Introduction:**

coronavirus disease (COVID-19) is a highly infectious disease caused by the novel coronavirus (SARS-CoV-2). Several public health and social protective measures that may prevent or slow down the transmission of COVID-19 were introduced. However, these measures are unfortunately being neglected or deliberately ignored by some individuals.

**Methods:**

a cross sectional online based survey was conducted to identify possible factors influencing public willingness to adhere to precautionary measures and preventive guidelines against COVID-19 during the lockdown periods in Sudan. The questionnaire was used to collect socio-demographic data of study participants, their health beliefs and willingness regarding adherence to precautionary measures against COVID-19 based on the constructs of the Health Belief Model.

**Results:**

a total of 680 respondents completed and returned the online questionnaire. Significant predictors of the willingness to adhere to the precautionary measures against COVID-19 were gender (β= 3.34, P<0.001), self-efficacy (β= 0.476, P<0.001), perceived benefits (β= 0.349, P<0.001) and perceived severity (β= 0.113, P=0.005). These factors explained 43% of the variance in respondents' willingness to adhere to COVID-19 precautionary measures. Participants who were female, confident in their ability to adhere to the protective measures when available, believing in the benefits of the protective measures against COVID-19 and perceiving that the disease could have serious consequences were more likely to be willing to adhere to the protective measures.

**Conclusion:**

female respondents and respondents having higher self-efficacy, higher perceived benefits and higher perceived severity were more likely to be willing to adhere to the protective measures against COVID-19 in Sudan.

## Introduction

Corona virus disease (COVID-19) is a highly infectious disease caused by the novel coronavirus (SARS-CoV-2) [1]. The virus was first detected in Wuhan, China [2] on December 2019. In the last two decades Coronaviruses have caused two large-scale pandemics, Severe Acute Respiratory Syndrome (SARS) [3] and Middle East respiratory syndrome (MERS) [4]. Several public health and social measures that may prevent or slow down the transmission of COVID-19 were introduced, including case isolation, identification and follow-up of contacts, adequate environmental disinfection, and use of personal protective equipment (PPE) [5]. By 27^th^ August 2020 the virus has reached 214 countries resulted in 24.2 million cases and 826000 confirmed deaths.

At that early stage of virus spread, there was no approved vaccine preventing corona virus disease. The best prevention was to avoid exposure to the virus [6]. In response to this pandemic, almost all countries imposed lockdown policy to slow the spread of the virus and introduced measures that may reduce the risk of exposure, these include: use of face masks; regular hand washing with soap or disinfection with hand sanitizer; avoidance of contact with infected people and complying with social distancing measures in crowded places; and avoid touching eyes, nose, and mouth with unwashed hands [7,8]. However, these guidelines, which were introduced to slow the spread of COVID-19 and contribute to public well-being, are unfortunately being neglected or deliberately ignored by some individuals [9]. Health Belief Model [10] was originally formulated to explain different preventive health behaviors. According to this model, the individual's health behavior depends on an individual's perception of being at risk to get the disease, perceived severity of the disease, perceived benefits and perceived costs of taking a particular health action [11] and feeling capable of implementing the desired behavior to achieve results [12]. This study aims to identify the possible factors influencing Sudanese people willingness to adhere to COVID-19 precautionary measures and preventive guidelines (staying at home during lockdown, wearing masks/gloves and following social distancing guidelines) using domains (constructs) of the Health Belief Model.

## Methods

**Study design and sampling:** a cross-sectional design was used to study participants´ intention to adhere to protective measures against COVID-19. The study was conducted in Sudan in 2020 during lockdown period. The study was conducted on 625 participants based on the assumption that willingness to adhere to protective measures =50%, precision =5% and alpha = 0.05. Convenience internet-based sampling technique was used.

**Data collection:** using Google forms, an online structured questionnaire was developed by the researchers based on review of previous literature. The questionnaire link was distributed to participants through social media platforms such as: Email, Facebook, WhatsApp, Twitter, etc. The questionnaire was used to collect socio-demographic data of study participants, their health beliefs and willingness to adhere to precautionary measures against COVID-19. The precautionary measures and preventive guidelines addressed in this study were: staying at home during lockdown periods, wearing masks/gloves and practicing social distancing on going out.

**Constructs of the Health Belief Model (HBM):** the scale measuring HBM constructs consists of 5 subscales measuring participants' perceived susceptibility and severity of COVID-19, perceived benefits and barriers to adhere to the precautionary measures and self- efficacy in adhering to preventive guidelines. Responses to scale items were scored on a five-point Likert scale ranging from 0 (strongly disagree) to 4 (strongly agree). The score was reversed for some items, it was calculated for each subscale, converted to percentage and categorized into high (> 66.67%), moderate (33.33% to 66.67%) and low (< or equal to 33.33%).

**Willingness of respondents to adhere to COVID-19 precautionary measures:** it was assessed using two statements: “In the coming period, I will try to stay at home and not go out unless necessary”, and “In the coming period, I will try to adhere to the protective measures (wearing a mask, gloves and practice social distancing) when going out”. Responses to scale items were scored on a five-point Likert scale ranging from 0 (strongly disagree) to 4 (strongly agree). The score was calculated and converted to percentage and categorized into high (> 66.67%), moderate (33.33% to 66.67%) and low (< or equal to 33.33%). The internal consistency of HBM subscales and the scale for willingness to adhere to precautionary measure were determined by Cronbach´s alpha coefficient. Cronbach´s alpha coefficients were 0.862 (perceived susceptibility), 0.798 (perceived severity), 0.799 (perceived benefits), 0.793 (perceived barriers), 0.757(self-efficacy) and 0.823 (willingness to adhere to precautionary measures).

**Statistical analysis of the data:** data were fed to the computer and analyzed using IBM SPSS software package version 20.0 (Armonk, NY: IBM Corp). Qualitative data were described using number and percent. Quantitative data were described using mean and standard deviation. Significance of the obtained results was judged at the 5% level. Pearson´s coefficient was used to correlate between the different variables used in the study. Linear regression analysis was performed to detect the significant predictors of the willingness to adhere to precautionary measures.

**Ethical considerations:** this study was approved by the Ethics Committee of the High Institute of Public Health, Alexandria University, Egypt in 2020. The questionnaire was preceded by a cover letter explaining the aim of the study followed by an invitation to participate. The anonymity and confidentiality of participants were guaranteed. Online submission of the questionnaire was considered as consent to participate in the study.

## Results

**Socio-demographic characteristics of the respondents:** a total of 680 Sudanese has responded to the questionnaire. The mean age of participants was 26.7 (±7.95 years), 56.8% of participants were females, 83.7% were living in Khartoum (the capital of Sudan), 79% were single and 77.6% were university undergraduates. The monthly income of about half the participants (49.6%) was just enough (Table 1).

**Table 1:** socio-demographic characteristics of study participants

Socio-demographic characteristics	Number	%
**Age**		
<20	58	8.5
20 - <40	566	83.2
40 - <60	52	7.6
60+	4	0.6
Mean ± SD	26.70 ± 7.95	
**Sex**		
Male	294	43.2
Female	386	56.8
**Residence**		
Northern State	16	2.4
Red Sea State	32	4.7
Al-Gezira State	51	7.5
Khartoum State	569	83.7
River Nile State	12	1.8
**Marital status**		
Single	537	79.0
Married	131	19.3
Divorced**/** Widowed	12	1.7
**Education**		
High school	35	5.1
University undergraduate	528	77.6
University graduate	117	17.2
**Income**		
Not enough	104	15.3
Just enough	337	49.6
Enough and saving	239	35.1

**Figure 1:**
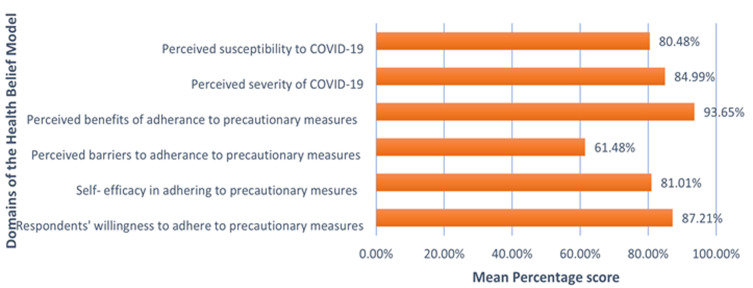
mean percentage score of the Health Belief Model domains and respondents' willingness to adhere to COVID-19 precautionary measures

**Mean percentage score of the Health Belief Model domains and respondents' willingness to adhere to COVID-19 precautionary measures:** the mean percentage score (MPS) of all the domains of the HBM -except for perceived barriers were pertinently high indicating that participants strongly believed in being susceptible to COVID-19 (80.48%) and that the disease could have severe consequences (84.99%). Participants also perceived that adherence to the protective measures such as social distancing and wearing masks was important and beneficial (93.65%) and were confident in their ability to adhere to these protective measures (81.01%) if they were available. Nevertheless, the MPS of perceived barriers was moderately high (61.48%) denoting that several barriers to the adherence to protective measures were reported by a significant proportion of participants. The MPS of willingness to adhere with authority guidelines was high (87.21%) reflecting participants´ strong will to adhere to the protective measures against COVID-19 (Figure 1).

**Respondents´ perceived susceptibility and severity (perceived threat) of COVID-19:** most of the participants agreed/strongly agreed on being at risk of getting COVID-19 (71.3%) and the possibility of getting infected when being in contact with a person not showing the symptoms of the disease (87.6%) meanwhile, they disagreed/strongly disagreed on the negative statement “COVID-19 disease is not transmitted through contact with surfaces or tools” (80.4%). However, more than one third (35.6%) of participants were not sure whether the hot climate in Sudan killed the virus and thus reduced the possibility of infection. More than 90% of the participants agreed/strongly agreed that COVID-19 is spreading rapidly and that it may lead to death (Table 2).

**Table 2:** respondents’ perceived susceptibility and severity (perceived threat) of COVID-19

HBM constructs and statements	Agree/SA		Neutral		Disagree/SD	
	N	%	N	%	N	%
**Perceived susceptibility**						
I think I am at risk of corona virus infection	485	71.3	150	22.1	45	6.6
I may become infected when in contact with a person who does not show symptoms of corona virus disease (COVID-19)	596	87.6	55	8.1	29	4.3
COVID-19 disease is not transmitted through contact with surfaces or tools	73	10.7	60	8.8	547	80.4
The hot climate in Sudan kills the virus and thus reduces the possibility of infection	85	12.5	242	35.6	353	51.9
The Sudanese citizen has strong immunity and therefore is less likely to contract the disease	85	12.5	127	18.7	468	68.8
**Perceived severity**						
The emerging corona virus is rapidly spreading	649	95.4	21	3.1	10	1.5
Coronavirus disease (COVID-19) may lead to death	631	92.8	27	4.0	22	3.2
Coronavirus disease is a common disease like the flu and hence it's not dangerous	97	14.3	68	10.0	515	75.7
I am not afraid of catching coronavirus disease	116	17.1	45	6.6	519	76.3

SA: Strongly agree, SD: Strongly disagree

**Respondents’ self-efficacy, perceived barriers and benefits of adherence to the precautionary measures against COVID-19:** more than 90% agreed/strongly agreed that adherence to protective measures reduced the number of cases and decreased spread of the disease. A great majority of the participants believed that they were capable of staying at home for the entire period of time specified by the ministry of health, and only go out if necessary (78.7%) and were able to commit to wearing the mask (if available) whenever they went out (91.6%). Regarding perceived barriers, going to bring the needs of the family was the most frequently reported factor hindering participants from staying at home (85.1%) followed by work requirements (62.4%) then feeling bored (46.8%), while disagreements between family members at home was the least mentioned barrier to staying at home (17.6%). Concerning factors hindering adherence to protective measures outside home, the absence of rules governing the distance between people in crowded places such as markets and means of public transportation was the barrier most agreed/strongly agreed upon (91.8%) followed by the unavailability and high prices of gloves/masks (76.3% and 72.6% respectively) (Table 3).

**Table 3:** respondents' self-efficacy, perceived barriers and benefits of adherence to precautionary measures against COVID-19

HBM constructs and statements	Agree/SA		Neutral		Disagree/SD	
	N	%	N	%	N	%
**Perceived barriers**						
**What are the factors that may hinder you from staying at your home?**						
Feeling bored	318	46.8	45	6.6	317	46.6
Work demands	424	62.4	49	7.2	207	30.4
Bring the needs of the family	579	85.1	16	2.4	85	12.5
Disagreements between family members at home	120	17.6	51	7.5	509	74.9
Feeling upset when staying at home	281	41.3	56	8.2	343	50.4
Feeling that I'm staying home indefinitely is ridiculous and illogical	158	23.2	45	6.6	477	70.1
**What are the factors that may hinder you from adhering to the preventive measures wearing a mask, gloves and social distancing when leaving the house?**						
I do not have masks/gloves	399	58.7	14	2.1	267	39.3
Sometimes I forget to wear a mask/glove or to keep an adequate distance between me and whomever I'm talking to	379	55.7	46	6.8	255	37.5
The absence of rules governing the distance between people in crowded places such as markets and public transportation	624	91.8	6	0.9	50	7.4
I feel embarrassed to wear the mask or keep a distance from whom I'm talking to	100	14.7	20	2.9	560	82.4
I do not think these measures are effective	72	10.6	45	6.6	563	82.8
High prices of masks/gloves	494	72.6	43	6.3	143	21.0
Face masks/gloves are not available in the market	519	76.3	83	12.2	78	11.5
I do not feel comfortable wearing the mask / glove	228	33.5	50	7.4	402	59.1
**Perceived benefits**						
Staying at home is important and essential	649	95.4	9	1.3	22	3.2
Adherence to preventive measures (wearing a mask, gloves and social distancing) when leaving home makes me feel safe.	645	94.9	22	3.2	13	1.9
Adherence to preventive measures helps in reducing the number of cases and controlling the spread of corona virus disease	666	97.9	9	1.3	5	0.7
**Self-efficacy**						
I can stay at home for the entire period specified by the ministry of health, and only go out if necessary	535	78.7	81	11.9	64	9.4
If I have gloves, I can stick to wearing them whenever I go out	583	85.7	57	8.4	40	5.9
If I have masks, I trust my ability to commit to wearing them whenever I go out	623	91.6	39	5.7	18	2.6
I find it difficult to adhere to social distancing, even in non-crowded places	278	40.9	75	11.0	327	48.1

SA: Strongly agree, SD: Strongly disagree, N; Number

**Correlation between constructs of the Health Belief Model and willingness of respondents to adhere to COVID-19 precautionary measures:** willingness to adhere to preventive guidelines was significantly correlated with all the parameters of HBM model. The five parameters of the model showed a significant positive correlation except for perceived barriers which, expectedly, showed a significant negative correlation with respondents´ willingness and with all other parameters of the HBM. Self-efficacy had the strongest positive correlation with the willingness to adhere to preventive measures (r =0.589) followed by perceived benefits (r =0.490). Intuitively, this means that the more confident the person is in his ability to perform the behavior (adhere to protective measures), the more likely he feels he would perform the behavior. However, willingness to adhere to protective measures is influenced by other factors as well including perceived benefits and perceived severity (Table 4).

**Table 4:** correlation between constructs of the Health Belief Model and willingness of respondents to adhere to COVID-19 precautionary measures

	P susc	P sev	P bar	P ben	SE	INT
Perceived susceptibility (P susc)	-					
Perceived severity (P sev)	0.452*	-				
Perceived barriers (P bar)	-0.331*	-0.336*	-			
Perceived benefits (P ben)	0.375*	0.372*	-0.258*	-		
Self-efficacy (SE)	0.210*	0.274*	-0.400*	0.456*	-	
Intention (INT)	0.229*	0.317*	-0.304*	0.490*	0.589*	-

r: Pearson´s coefficient *: Statistically significant at p ≤ 0.05

**Predictors of respondents´ willingness to adhere to COVID-19 precautionary measures:**Table 5 shows the multivariate linear regression analysis of respondents' willingness to adhere to the precautionary measures, with some independent variables. After adjustment for all possible confounders, it was found that the significant predictors of respondent´s willingness to adhere to protective measures were gender (β=3.34, P<0.001), self-efficacy (β= 0.476, P<0.001), perceived benefits (β= 0.349, P<0.001) and perceived severity (β= 0.113, P=0.005). These factors explained 43% of the variance in participants´ willingness. Participants who were female, confident in their ability to adhere to the protective measures when available, believing in the benefits of the protective measures against COVID-19 and perceiving that the disease could have serious consequences were more likely to be willing to adhere to the protective measures (Table 5).

**Table 5:** summary of multivariate linear regression analysis for the parameters affecting respondents' willingness to adhere to COVID-19 precautionary measures

Parameters	#Multivariate	
	p	B (95%C. I)
Sex (1=male/ 2= female)	<0.001*	3.342 (1.592 - 5.091)
Income	0.196	0.858 (-0.443 - 2.159)
Perceived susceptibility	0.489	0.027 (-0.104 - 0.050)
Perceived severity	0.005*	0.113 (0.035 - 0.192)
Perceived barriers	0.730	-0.013 (-0.087-0.061)
Perceived benefits	<0.001*	0.349 (0.250 - 0.449)
Self-efficacy	<0.001*	0.476 (0.400 - 0.552)

R2=0.434, F=73.522*,p<0.001*

## Discussion

This study focused on identifying the health beliefs influencing willingness to adhere to precautionary behavior against the novel corona virus pandemic in Sudan. While suffering the burdens of civil war, political instability and economic meltdown [13]. Sudan, like all other countries, was hit by the COVID-19 pandemic. Despite the unfavorable political and economic conditions and to our surprise the respondents showed high willingness to adhere to the protective measures against COVID-19. Most of the respondents were relatively young which offers some explanation to their readiness to accept the change and fight their battle against COVID-19. Evidently, females were more likely to be willing to adhere to the protective measures against the disease. This finding was supported by several studies [14-18] which all agreed that females were more compliant to health-related guidelines than males. Intuitively, health educators should invest more effort in educating females who would, in turn, act as change agents influencing their social networks.

Regarding health beliefs, participants scored high on their health beliefs about adherence to the protective measures. It seems that the worldwide awareness-raising campaigns played an important role in increasing peoples´ knowledge of the virus and helped them acquire a more negative attitude towards the disease and a more positive attitude towards adopting the protective measures against the disease. Hence, most participants in the present study believed in being susceptible to the disease whether directly through their contact with infected people or indirectly through their contact with virus-contaminated surfaces or tools. However, a non-negligible proportion of participants neither agreed nor disagreed that the hot climate in Sudan killed the virus and believed that the Sudanese citizen had strong immunity and therefore were less likely to contract the disease, without documented scientific evidence. Such findings which have been previously reported [13] should draw the attention of health authorities in Sudan to the need of providing credible information to correct the misunderstanding and clear the ambiguity people might have about the disease. Nonetheless, it seems that the role of perceived susceptibility was not very evident in enhancing participants´ intention to adhere to the protective measures.

An intriguing result was that among health beliefs, the self-efficacy and not perceived barriers, was the most significant predictor of respondent´s willingness to adhere to protective measures. Feeling capable of adhering to protective measures against COVID-19 has strongly influenced participants´ willingness to do so. This finding highlights the importance of enhancing people´s confidence in their ability to adhere to protective measures by disseminating credible information about these measures: what they are, where to be found, when and how to be used. Moreover, they should be affordable and accessible. Additionally, enforcing rules concerning social distancing and wearing masks would transform these precautionary measures into accepted social norms, thus, encouraging people and giving them the confidence to adhere to such measures. In his Social Cognitive Theory (SCT), Albert Bandura described self-efficacy as the most important pre-requisite for behavior change [19,20]. Indeed, a considerable amount of research revealed that self-efficacy was a significant predictor of health behavior [21-23] lending support to our findings. Examination of correlation analysis revealed that respondents´ willingness to adhere to the protective measures was also influenced by perceived benefits of the protective measures and perceived severity of the disease, in the mentioned order. These findings should direct our efforts to the priority areas needed to be addressed while educating people about the importance of adherence to the protective measures. Undoubtedly, knowing the role of each protective measure in protecting people from being infected by the novel coronavirus and from the possible serious consequences of the disease would encourage them to adhere to these protective measures. Previous research has shown perceived benefits and perceived severity to be significant predictors of preventive health behavior [24-27].

Several barriers to adhere with precautionary behavior were documented in the current study, most important of which were the high prices of masks/gloves, their unavailability in the market and the absence of rules governing the distance between citizens in public places and public transportation. As regards staying at home, the most reported constraints were bringing family needs and work demands. Despite not being a significant predictor of respondents´ willingness, these barriers should not be overlooked. In fact, resolving these barriers would be an avenue to enhancing people´s self-efficacy and perceived controllability of adherence behavior. Ultimately, emphasis on some HBM constructs does not mean that we should ignore other constructs to achieve synergetic effect. In contradiction to our findings, several studies found perceived barriers to be a significant predictor of preventive health behaviors including immunization and screening behaviors [28-30].

**Implications for research and practice:** this research could be an important guide in designing health education messages to enhance adherence to the protective measures against COVID-19 especially in low- and middle-income countries. Educational messages should essentially focus on improving people´s self-efficacy in adhering to the protective measures. Moreover, more efforts should be invested in targeting females who would act as influential change agents among their social networks.

**Limitations:** cues to action were not assessed in this study to avoid a long questionnaire. However, knowing the triggers instigating participants to adhere to the protective measures would have certainly added to this work. We used convenience sampling technique; hence the generalization of results would not be possible. Nonetheless, the present research was able to shed important light on some of the factors influencing participants´ willingness to adhere to the protective measures against COVID-19 in Sudan. A subjective tool -self-report questionnaire -was used to collect data; therefore, participants´ responses might be affected by social desirability.

## Conclusion

Despite the unfavorable political and economic conditions in Sudan, study respondents showed high willingness to adhere to the precautionary measures and health authority guidelines against COVID-19. Female respondents and respondents having higher self-efficacy, higher perceived benefits and higher perceived severity were more likely to be willing to adhere to the protective measures against COVID-19 in Sudan. The Health Belief Model was a useful tool in predicting the beliefs affecting respondent's willingness to adhere to COVID-19 precautionary measures.

### What is known about this topic

- COVID-19 pandemic has posed unprecedented challenges and impact on all aspects of human life;
- Protective measures and precautionary guidelines against COVID-19 are being neglected by some individuals in the community;
- The Health Belief Model has been applied widely to explain different preventive health behaviors.

### What this study adds

- Going to bring the needs of the family was the most frequently reported factor hindering respondents from staying at home during lockdown periods in Sudan followed by work requirements and feeling bored;
- Absence of rules governing the distance between people in crowded places such as markets and means of public transportation, unavailability and high prices of gloves/masks were the most reported barriers hindering respondents from adhering to precautionary measures outside home;
- Based on our findings, health education messages regarding COVID-19 should essentially focus on improving people’s self-efficacy in adhering to protective measures and precautionary guidelines against COVID-19.
